# Virtual Screen for Repurposing of Drugs for Candidate Influenza a M2 Ion-Channel Inhibitors

**DOI:** 10.3389/fcimb.2019.00067

**Published:** 2019-03-26

**Authors:** Draginja Radosevic, Milan Sencanski, Vladimir Perovic, Nevena Veljkovic, Jelena Prljic, Veljko Veljkovic, Emily Mantlo, Natalya Bukreyeva, Slobodan Paessler, Sanja Glisic

**Affiliations:** ^1^Center for Multidisciplinary Research, Institute of Nuclear Sciences VINCA, University of Belgrade, Belgrade, Serbia; ^2^Biomed Protection, Galveston, TX, United States; ^3^Department of Pathology, University of Texas Medical Branch, Galveston, TX, United States; ^4^Institute for Human Infections and Immunity, University of Texas Medical Branch, Galveston, TX, United States

**Keywords:** influenza A, IAV matrix protein 2, drug repurposing, virtual screening, drug resistance

## Abstract

Influenza A virus (IAV) matrix protein 2 (M2), an ion channel, is crucial for virus infection, and therefore, an important anti-influenza drug target. Adamantanes, also known as M2 channel blockers, are one of the two classes of Food and Drug Administration-approved anti-influenza drugs, although their use was discontinued due to prevalent drug resistance. Fast emergence of resistance to current anti-influenza drugs have raised an urgent need for developing new anti-influenza drugs against resistant forms of circulating viruses. Here we propose a simple theoretical criterion for fast virtual screening of molecular libraries for candidate anti-influenza ion channel inhibitors both for wild type and adamantane-resistant influenza A viruses. After *in silico* screening of drug space using the EIIP/AQVN filter and further filtering of drugs by ligand based virtual screening and molecular docking we propose the best candidate drugs as potential dual inhibitors of wild type and adamantane-resistant influenza A viruses. Finally, guanethidine, the best ranked drug selected from ligand-based virtual screening, was experimentally tested. The experimental results show measurable anti-influenza activity of guanethidine in cell culture.

## Introduction

Influenza is a serious global public health concern. Regardless of the availability of antiviral drugs and vaccines, according to the World Health Organization's estimates, influenza is the cause of 3 to 5 million cases of severe illness and about 290,000–650,000 deaths in seasonal outbreaks worldwide (WHO Influenza, 2018). Annual “flu” vaccination has a primary role in preventing influenza A and B virus infections and increasing population immunity even though the efficacy of the seasonal flu vaccines may vary from year to year (Bridges et al., 2013). Since the current flu vaccination approach is imperfect, a substantial portion of the population is susceptible to infection even after vaccination every year. Therefore, alternative strategies should be considered to improve our therapeutic abilities for those patients that develop clinical flu. This would be especially important in the pandemic setting with rapid virus transmission due to the currently limited ability for fast, new vaccine production (Bridges et al., 2013). Current treatment and prophylaxis against seasonal influenza is limited to the only licensed class of antivirals, namely neuraminidase inhibitors (NAIs). Oseltamivir and zanamivir are currently licensed worldwide while peramivir and laninamivir are approved in some countries (Ison, 2017). The frequency of NAI resistance in currently circulating strains is low, <1% (Hurt et al., 2016), but resistance to oseltamivir, the most widely used NAI, was extensive amongst former seasonal H1N1 viruses in 2008. (Hurt et al., 2009), and was detected in localized clusters of oseltamivir-resistant H1N1pdm09 (Hurt et al., 2011). Nevertheless, the therapeutic window for treatment with these drugs is very short and patients benefit the most when treated 24–48 h after the onset of “flu” symptoms (Ison, 2017).

The first of the two classes of FDA-approved anti-influenza drugs are adamantanes, amantadine and rimantadine, that inhibit viral replication by blocking the wild-type (WT) M2 proton channel.

IAV matrix protein 2 (M2), an ion channel protein, is one of the most conserved viral proteins and essential for efficient virus replication, and is thus an important anti-influenza drug target (Takeda et al., 2002). Matrix protein 2 (M2) is a 97-residue-long viral protein that encompasses a 19-residue-long hydrophobic transmembrane domain (TM) that forms a homotetrameric proton-selective channel involved in proton conductance and drug binding (Lamb et al., 1985; Sakaguchi et al., 1997). Adamantanes have been used successfully against influenza A virus infection for more than 30 years because of their wide accessibility and low price (Dolin et al., 1982). However, as a consequence of the lack of activity against influenza B (Mould et al., 2003), adverse effects, and the rapid emergence of resistance during treatment or even in the absence of selective drug pressure, the Centers for Disease Control and Prevention (CDC) have strongly recommended against the use of this class of drugs (CDC, 2006). The molecular basis for resistance to adamantanes is connected with several amino acid substitutions and the M2-S31N variant is found in more than 95% of the currently circulating influenza A viruses (Dong et al., 2015). The expansion of M2 viruses with S31N mutation in the early 2000s is not a consequence of drug selection pressure but is connected to advantageous substitutions elsewhere in the virus, in a process denoted as genetic “hitch-hiking” (Simonsen et al., 2007). On the other hand, the latest report of M2 S31 and D31 viruses in Australia suggests that the role of the M2 N31 residue in viral fitness is no longer as important as it used to be (Hurt et al., 2017). Considering all these facts, new effective anti-influenza M2 inhibitors that target both WT and S31N mutant are greatly needed. Several high-resolution M2 structures that provide important insights into the favorable structural features can be employed for designing new M2 inhibitors (Hong and DeGrado, 2012).

A recent, and very popular drug discovery approach—drug repurposing (DR), wherein old drugs are given new indication by exploring new molecular pathways and targets for intervention (Strittmatter, 2014)- offers potential economic advantage and shorter regulatory process for the clinical approval. The continuous increase of drug-resistant pathogens is a great challenge for treatment of infectious diseases and DR serves as an alternative approach for rapid identification of effective therapeutics (Zheng et al., 2018). Drug repurposing (DR) applied to viral infectious diseases integrates both screening of bioactive small-molecule collections and computational methods to find a molecule, a pathway, or a biological activity that could be used against the virus of interest (Mercorelli et al., 2018). Two clinical trials against influenza viruses with repositioned drugs are currently underway: (1) The first trial (phase 2b/3clinical trial) combines clarithromycin and naproxen along with oseltamivir in a triple-drug combination and; (2) the second trial is focused on testing efficacy of an antiparasitic drug, nitazoxanide, against influenza viruses (Phase III) (Mercorelli et al., 2018).

In this study we propose a simple theoretical criterion for fast virtual screening of molecular libraries for candidate anti-influenza M2 ion channel inhibitors both for wild type and adamantane-resistant influenza A viruses. After *in silico* screening of drug space using the EIIP/AQVN filter, and further filtering of drugs by ligand based virtual screening and molecular docking, we proposed the five best candidate drugs as potential dual inhibitors of wild type and adamantane-resistant influenza A viruses.

## Materials and Methods

For screening of drugs for repurposing to select candidates for influenza M2 inhibitors, 2,627 approved small molecule drugs from DrugBank (http://www.drugbank.ca) were screened. To define the predictive criterion for the selection of Influenza M2 candidates, the learning set (Supplementary Tables 1, 2) was composed of all active compounds from ChEMBL Target Report Card (https://www.ebi.ac.uk/chembl/target/inspect/CHEMBL613740) (EMBL-EBI. ChEMBL). (EMBL-EBI. ChEMBL. Available online: https://www.ebi.ac.uk/chembl/ (accessed on June 30, 2018) against influenza A virus M2 (Target ID CHEMBL613740) both for wild type (WT) and S31N, with corresponding IC50 values. The total number of reported compounds for WT and S39N of M2 channel were 50 and 49, respectively. After removal of duplicates and inactive compounds, the final number of compounds was 15 for WT and 12 for the S31N mutant (Supplementary Tables 1, 2). The control data sets were compounds from PubChem compounds database (http://www.ncbi.nlm.nih.gov/pccompound).

### Virtual Screening

The virtual screening (VS) protocol included the application of subsequent filters to select candidate dual inhibitors of M2 ion channel. The first EIIP/AQVN filter approach was employed for *in silico* screening of the ChEMBL Target Report Card (https://www.ebi.ac.uk/chembl/target/inspect/CHEMBL613740) and DrugBank (http://www.drugbank.ca) (Wishart et al., 2006) and then proceeded by ligand-based screening.

### EIIP/AQVN

The EIIP for organic molecules can be determined by the following simple equation derived from the “general model pseudopotential (Veljkovic et al., 2011).

(1) $$\begin{array}{l} \text{EIIP}=0.25{\text{Z}}^{*}\text{sin}\left(1.04\text{π}{\text{Z}}^{*}\right)/2\text{π} \end{array}$$

where Z^*^ is the average quasi valence number (AQVN) determined by

(2) $$\begin{array}{l} {\text{Z}}^{*}=\sum \text{m}\left(\text{niZi}/\text{N}\right) \end{array}$$

Where *Zi* is the valence number of the *i*th atomic component, *ni* is the number of atoms of the *i*th component, *m* is the number of atomic components in the molecule, and *N* is the total number of atoms. EIIP values calculated according to Equations (1, 2) are expressed in Rydberg units (Ry).

### Ligand-Based Virtual Screening

To screen selected compounds from Drugbank, both learning set compounds and candidates from the previous step were converted to 3D sdf format from smiles. GRIND descriptors of molecules were calculated, based on molecular interaction field (MIF) probes (Duran et al., 2009). Computation method for descriptor generation was GRID with step 0.5. Applied probes (mapped regions of molecule surface) were DRY (hydrophobic interactions) O (hydrogen bond acceptor) N1 (hydrogen bond donor) and TIP (molecular shape descriptor). Discretization Method was AMANDA (Duran et al., 2008), with scale factor 0.55. Cut off was set to: DRY −0.5 O −2.6 N1 −4.2 TIP −0.75. Encoding Method was MACC2 and weights were the following: DRY: −0.5, O: −2.6, N1: −4.2, TIP: −0.75. Number of PCA components was set to five. Explained variance of such obtained model was 58.84%. Then, learning set compounds were imported and served for screening the candidate compound database. All calculations were carried in Pentacle software version 1.06 for Linux (Pastor et al., 2000).

### Molecular Docking

#### Receptor Preparation

Crystal structures of the wild type M2 channel and the S31N mutant channel were downloaded from RCSB PDB database (https://www.rcsb.org/) with PDBIDs 2KQT (Cady et al., 2010) and 2LY0 (Wang et al., 2013) respectively. All ligands, ions and water molecules were removed from structures. All hydrogen atoms were added on protein structures and then truncated to only polar hydrogen atoms during the preparation process. The receptor was prepared in ADT Tools 1.5.6 (Sanner, 1999; Morris et al., 2009).

#### Ligand Preparation

Ligands were converted from 3Dsdf to mol2 format and imported to Avogadro software in order to protonate them at physiological pH. Molecules were prepared for MOPAC 2016 (Stewart, 2016) and geometrically optimized on PM7 (Stewart, 2013) level of theory. They were further prepared for molecular docking in ADT Tools.

### Molecular Docking

A grid box with dimensions 24 × 24 × 24 A was placed in the center of the binding site of the protein receptor. Exhaustiveness was set to 50. Molecular docking was carried in Autodock Vina (Trott and Olson, 2010).

### *In vitro* Efficacy Testing of Guanethidine Against Influenza a (h1n1) Virus

Influenza A/CA/07/2009 (H1N1) virus was premixed with 1, 10, and 100 μM of guanethidine and incubated at 37C for 1 hr. Positive control wells were prepared by mixing influenza A/CA/07/2009 (H1N1) virus with 10 μM of merimepodib. MDCK cells were then infected in triplicates with influenza A/CA/07/2009 (H1N1) virus / drug mixture. After ~1 h of incubation at 37°C and 5% CO2, cells were washed with serum free media and 1 × of each compound dose was added to the cells. Virus control wells as well as untreated control wells were included in triplicates. Cells were incubated at 37C and 5% CO2 and samples were collected at 0, 1, 2, and 3 days post-infection. Samples were stored at −80°C until the day of analysis. The influenza virus titer in MDCK cells via TCID50 was performed for each sample collected at days 0, 1, and 2 post-infection.

## Results

**Figure 1:**
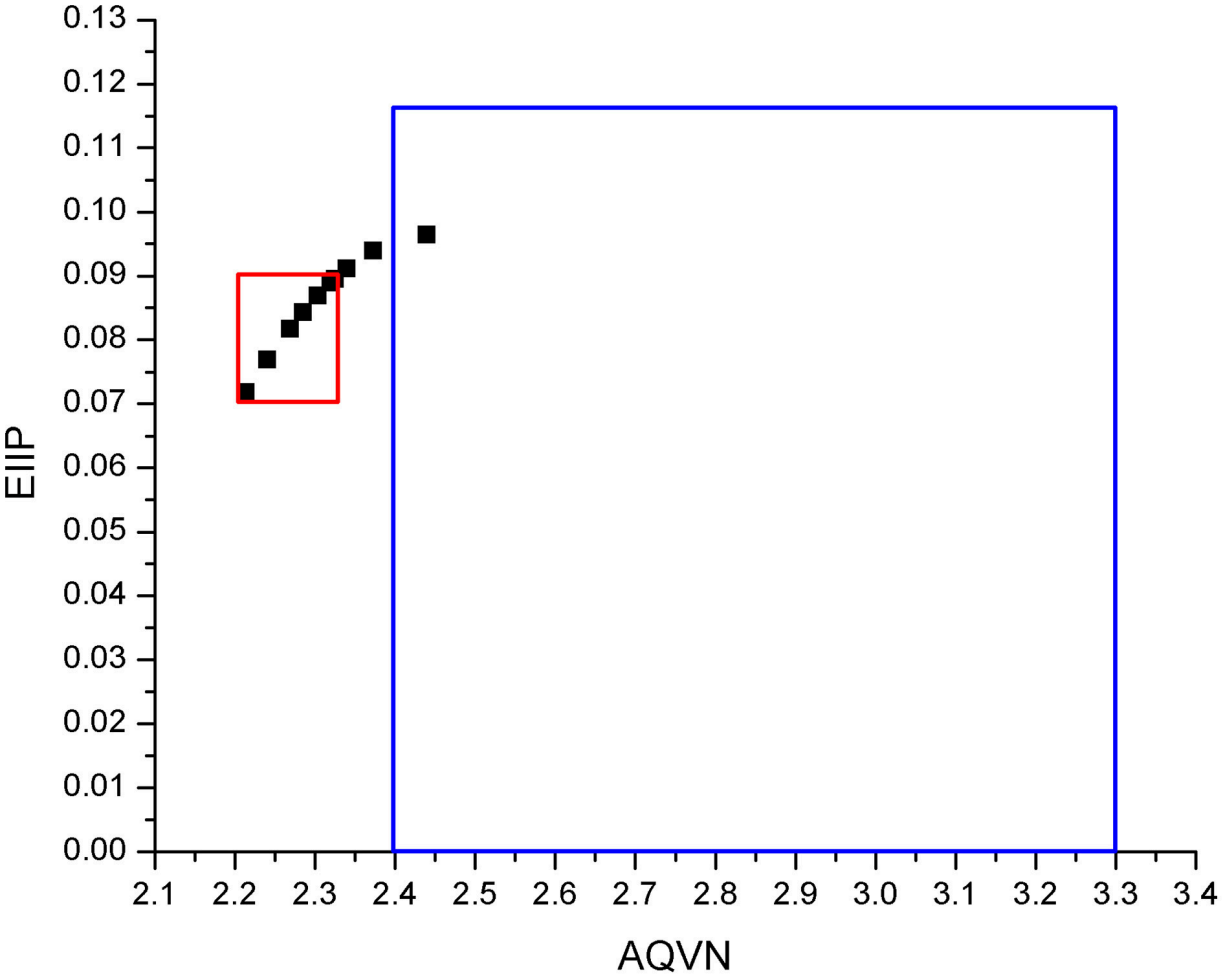
Schematic presentation of the EIIP/AQVN criterion for selection of candidate M2 inhibitors. Common domain of active compounds for both WT and S31N M2 (red) with AQVN (2.21–2.32), EIIP (0.071–0.089). Chemical space (blue) with AQVN (2.40–3.30) EIIP (0.000–0.116)—EIIP/AQVN domain of homologous distribution of >90% compounds from PubChem Compound Database.

The virtual screening (VS) protocol in this study was based on the application of sequential filters to select candidate dual inhibitors of the M2 ion channel. Previously it was shown for molecular targets in diverse pathological states that small molecules with similar AQVN and EIIP values interact with the common therapeutic targets (Veljkovic et al., 2011, 2013). This resulted in determining criteria for virtual screening of molecular libraries for compounds with similar therapeutic properties (Veljkovic et al., 2013). The learning set consists of M2 WT (Supplementary Table 1), and M2 S31N mutant (Supplementary Table 2) ion channel inhibitors from the ChEMBL Target Report Card (https://www.ebi.ac.uk/chembl/target/inspect/CHEMBL613740) (EMBL-EBI. ChEMBL). The AQVN/EIIP descriptor values were calculated for the learning set (Figure 1) and range for selection was based on their distribution. AQVN descriptor values were in range 2.21–2.32 for WT and 2.21–2.44 for S31N mutant. More than 80% of the compounds of WT inhibitors and 83% M2 S31N mutant ion channel inhibitors from the learning set were inside the common active domain for both while having AQVN and EIIP values within the intervals of (2.21–2.32) and (0.071–0.089). Inside this common active domain is also amantadine with AQVN/EIIP 2.214/0.0717. The reported domain was selected as a criterion for the selection of compounds representing candidate dual M2 WT and S31 mutant ion channel inhibitors (Figure 1). By applying the EIIP/AQVN-based virtual screening criterion, 39 drugs were chosen (Table 1) out of 2,627 approved drugs from the DrugBank (http://www.drugbank.ca) (Wishart et al., 2006).

**Table 1:** Approved drugs screened for candidate anti-M2 inhibitors.

**Drugbank accession number**	**Name**	**Chemical formula**	**AQVN**	**EIIP**
DB00915	Amantadine	C10H17N	2.214286	0.071739
DB06689	Ethanolamine Oleate	C18H34O2.C2H7NO	2.215385	0.071958
DB00153	Ergocalciferol	C28H44O	2.219178	0.072708
DB00898	Ethanol	C2H6O	2.222222	0.073303
DB01105	Sibutramine	C17H26ClN	2.222222	0.073303
DB01158	Bretylium	C11H17BrN	2.233333	0.075425
DB00804	Dicyclomine	C19H35NO2	2.245614	0.077673
DB00146	Calcidiol	C27H44O2	2.246575	0.077845
DB01436	Alfacalcidol	C27H44O2	2.246575	0.077845
DB00154	Dihomo-Î^3^-linolenic acid	C20H34O2	2.25	0.078451
DB00592	Piperazine	C4H10N2	2.25	0.078451
DB01191	Dexfenfluramine	C12H16F3N	2.25	0.078451
DB01431	Allylestrenol	C21H32O	2.259259	0.080048
DB00375	Colestipol	C8H23N5.C3H5ClO	2.26087	0.080319
DB00330	Ethambutol	C10H24N2O2	2.263158	0.080701
DB00162	Vitamin A	C20H30O	2.27451	0.082539
DB01365	Mephentermine	C11H17N	2.275862	0.082751
DB01170	Guanethidine	C10H22N4	2.277778	0.08305
DB00132	Alpha-Linolenic Acid	C18H30O2	2.28	0.083392
DB06809	Plerixafor	C28H54N8	2.288889	0.084723
DB08868	Fingolimod	C19H33NO2	2.290909	0.085017
DB00858	Drostanolone	C20H32O2	2.296296	0.085784
DB00136	Calcitriol	C27H44O3	2.297297	0.085924
DB00910	Paricalcitol	C27H44O3	2.297297	0.085924
DB00376	Trihexyphenidyl	C20H31NO	2.301887	0.086554
DB01022	Phylloquinone	C31H46O2	2.303797	0.086811
DB00191	Phentermine	C10H15N	2.307692	0.087326
DB00313	Valproic Acid	C8H16O2	2.307692	0.087326
DB01577	Methamphetamine	C10H15N	2.307692	0.087326
DB06204	Tapentadol	C14H23NO	2.307692	0.087326
DB06709	Methacholine	C8H18NO2	2.310345	0.087669
DB08887	Icosapent ethyl	C22H34O2	2.310345	0.087669
DB01187	Iophendylate	C19H29IO2	2.313725	0.088098
DB01337	Pancuronium	C35H60N2O4	2.316832	0.088483
DB00947	Fulvestrant	C32H47F5O3S	2.318182	0.088648
DB01083	Orlistat	C29H53NO5	2.318182	0.088648
DB00387	Procyclidine	C19H29NO	2.32	0.088868
DB00942	Cycrimine	C19H29NO	2.32	0.088868
DB08804	Nandrolone decanoate	C28H44O3	2.32	0.088868

All 39 selected drugs were imported in Pentacle software, protonated at pH 7.4, and aligned toward principal moment of inertia. In ligand based virtual screening, we used centroid distance method as criteria for similarity between learning set and candidate compounds. Top 5 candidates from DrugBank selection are presented in Table 2.

**Table 2:** Five best candidates from virtual screening, with Drugbank ID, Similarity distance, structure, and EIIP descriptor values.

**Drugbank ID**	**Name**	**Similarity distance from centroid**	**Structure**	**AQVN**	**EIIP**
DB01170	Guanethidine	1.3446	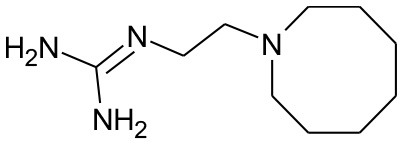	2.277778	0.08305
DB00191	Phentermine	1.4234	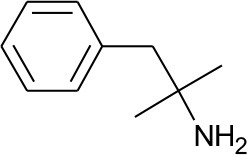	2.307692	0.087326
DB01577	Methamphetamine	1.4334	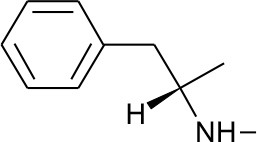	2.307692	0.087326
DB01191	Dexfenfluramine	1.5377	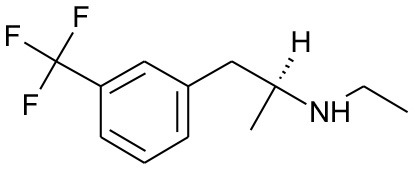	2.25	0.078451
DB00942	Cycrimine	1.6057	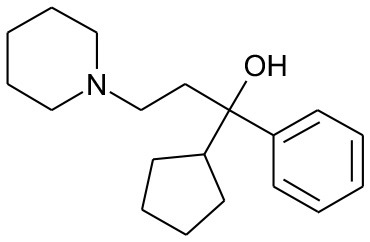	2.32	0.088868

**Figure 2:**
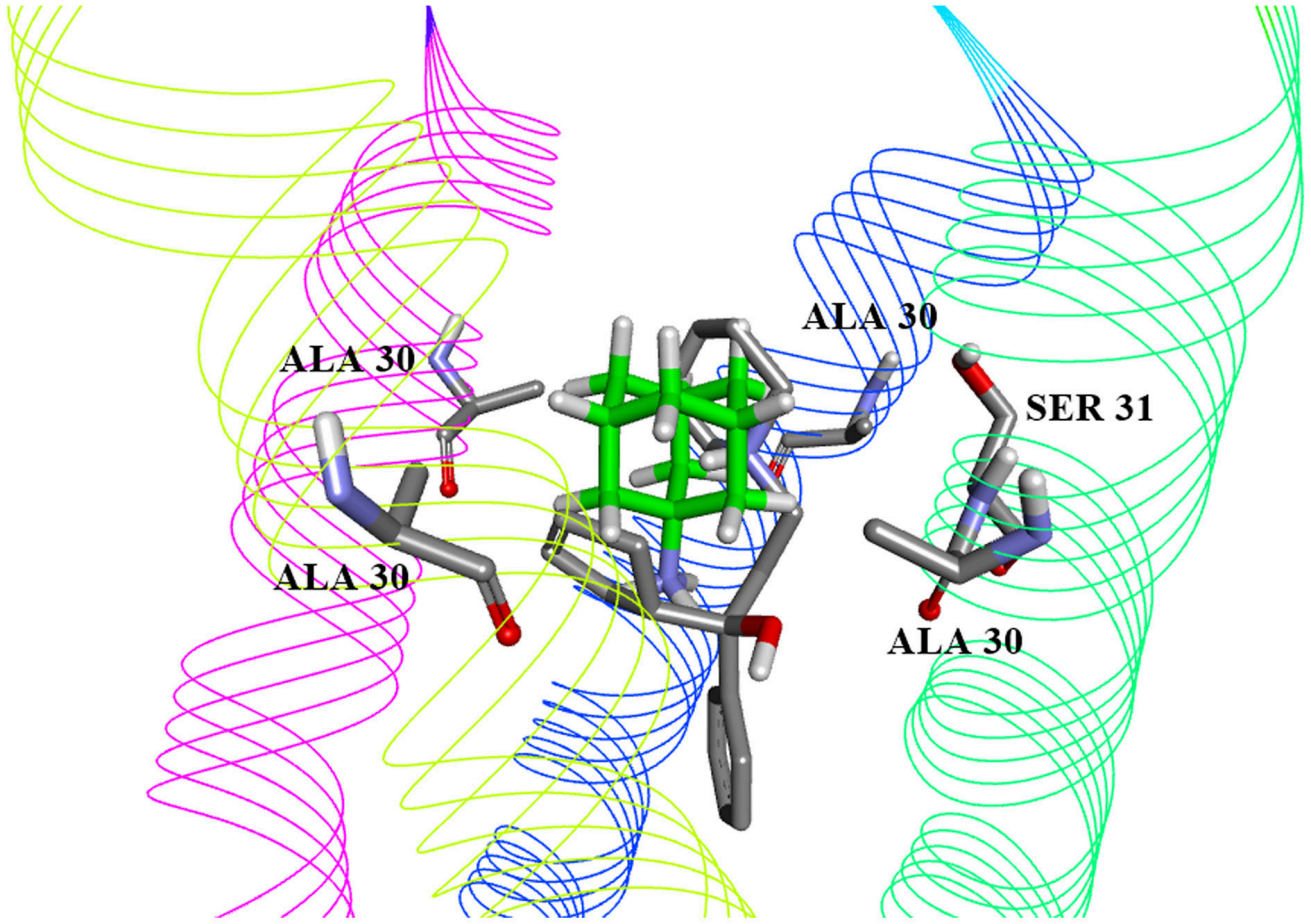
Best-ranking docked conformation of Cycrimine (gray carbon atoms) in solid state NMR structure of WT M2 channel (PDB 2KQT), compared to amantadine coordinates in complex (green carbon atoms).

**Figure 3:**
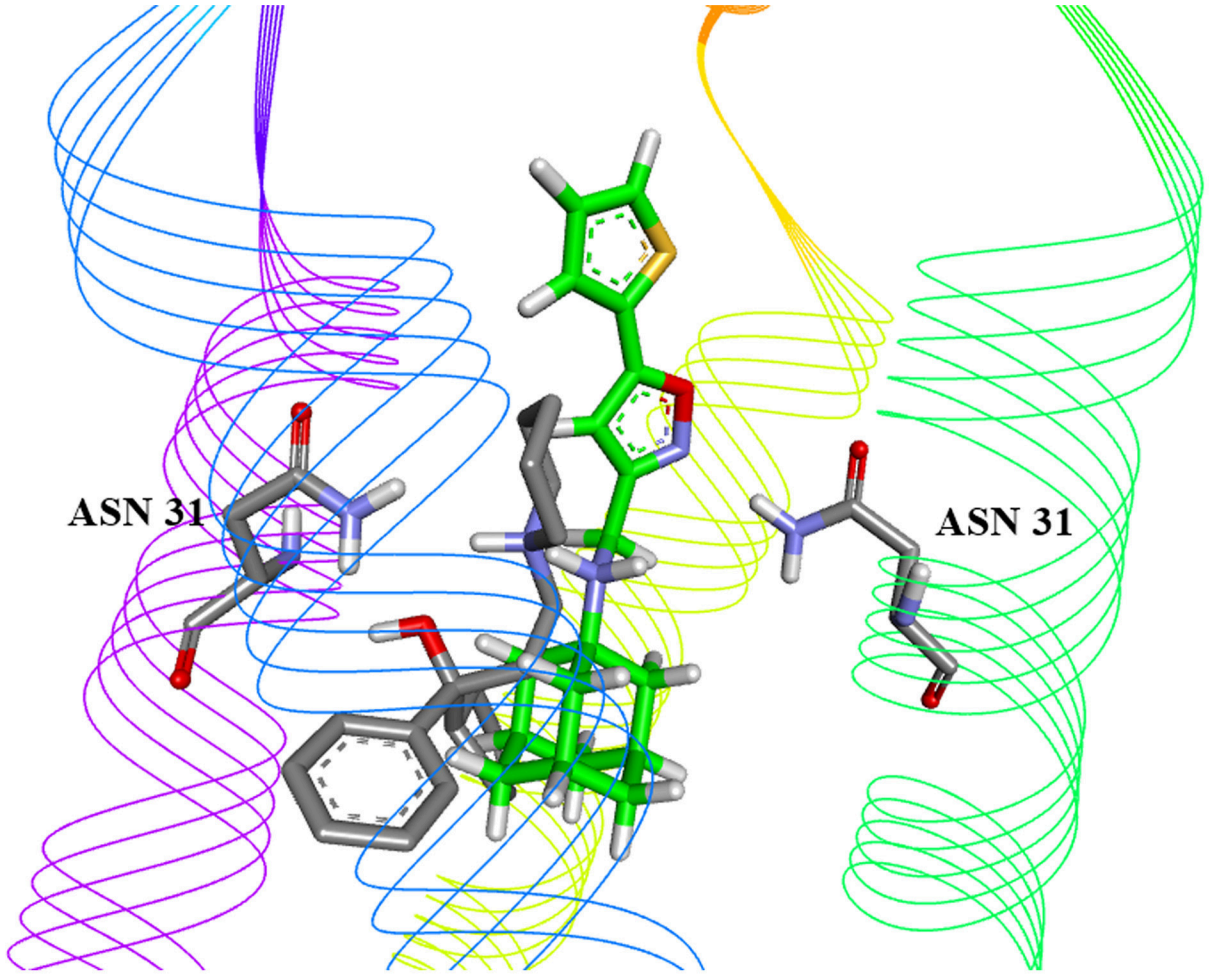
Best-ranking docked conformation of Cycrimine (gray carbon atoms) in solid state NMR structure of S31N mutant M2 channel (PDB 2LY0), compared to drug M2WJ332 coordinates in complex (green carbon atoms).

We further carried out molecular docking of five candidates to both the wild type M2 channel and S31N mutant channel. The docking energies obtained are presented in Table 3. The candidate with the lowest binding energy and equal affinity to both WT channel and S31N mutant channel was cycrimine, with docking energy −8.3 kcal/mol. Docked conformations of cycrimine are presented on Figures 1, 2. In both WT and S31N mutant of M2 channel, cycrimine conserves corresponding intermolecular receptor-ligand interactions, Ala 30 and Ser 31 in case of WT and Asn 31 in the case of the S31N mutant. As presented on Figures 2, 3, the orientation of cycrimine, compared to crystal coordinates of amandatine and M2WJ332 show cycrimine's ability to form hydrogen bond interactions with Ser 31 i.e., Asn 31, while keeping hydrophobic interactions with Ala 30. This could be a possible explanation as to why cycrimine shows relatively high and equal affinity to both WT and S31N mutant M2 channels (−8.3 kcal/mol, or 800 nM). Other compounds show similar binding patterns as cycrimine; however, in most other cases the affinity ratio is in favor of the WT M2 channel protein.

**Table 3:** Docking energies of five best candidates from virtual screening, with Drugbank ID, Docking energies, and affinity ratio.

**Drugbank ID**	**Name**	**Docking energy on WT M2 channel (kcal/mol)**	**Docking energy on S31N mutant M2 channel (kcal/mol)**	Affinity ratio WT:S31N^*^
DB00942	Cycrimine	−8.3	−8.3	1
DB01191	Dexfenfluramine	−6.3	−6.0	0.6
DB01170	Guanethidine	−5.9	−5.7	0.71
DB00191	Phentermine	−5.3	−4.8	0.43
DB01577	Methamphetamine	−4.8	−4.5	0.6

* *$Ratio=\frac{K_{1}}{K_{2}}=exp\left(\frac{\left(\Delta G_{1}-\Delta G_{2}\right)}{RT}\right)$*.

In order to validate our *in silico* screening approach, we examined the antiviral activity of guanethidine, the top candidate from the DrugBank selection (Table 2), *in vitro*. As a positive control, influenza virus was premixed with 10 uM of merimepodib, an IMPDH inhibitor with known antiviral activity against a variety of viruses including influenza (Markland et al., 2000; Tong et al., 2018). Addition of guanethidine to cells infected with 2009 H1N1 pandemic influenza virus resulted in significantly lower viral titers in a dose-dependent manner. Both 100 and 10 μM guanethidine treatment resulted in significant reductions in viral titers at day 1 post-infection, with 100 μM of guanethidine producing a 1–2 log reduction in viral titers.

## Discussion

Current prevention and treatment options for influenza A and B infections are insufficient due to increased clinical use of licensed antivirals leading to the emergence of resistant viral strains (Hayden and de Jong, 2011). In a quest for new preventive and therapeutic options to minimize drug resistance and threats of outbreaks of pandemic viruses, the main obstacle is the fact that drug development is an expensive, time-consuming, and risky enterprise. Therefore, drug repurposing represents a promising therapeutic strategy for many viral diseases including anti-influenza A and B treatment. Various predictive computational approaches have been developed to identify drug repositioning opportunities against influenza viruses (Sencanski et al., 2015). Previously, the EIIP/AQVN criterion has been proven to be an efficient filter in virtual screening of molecular libraries for candidate inhibitors of HIV and Ebola virus infection (Tintori et al., 2007; Veljkovic et al., 2015a,b). Using this approach, ibuprofen was selected as an inhibitor of the Ebola virus infection, and this prediction was later experimentally confirmed (Zhao et al., 2016; Paessler et al., 2018.)

To select drug candidates for M2 inhibitors, the virtual screening protocol in our study was based on the application of successive filters. The previous study of EIIP/AQVN distribution of compounds from the PubChem database (http://www.ncbi.nlm.nih.gov/pccompound) showed that 92.5% compounds from PubChem are homogenously distributed inside EIIP and AQVN intervals (0.00–0.11 Ry) and (2.4–3.3), respectively (Veljkovic et al., 2011). The domain that encompasses the majority of known chemical compounds was designated as a “basic EIIP/AQVN chemical space” (BCS). Results of the application of VS based on the EIIP/AQVN filter in this study showed that the active group of candidate M2 inhibitors is very specific, belonging to the sparse cluster of compounds that are out of BCS. This finding indicates that testing only a minor fraction of the compounds from the active EIIP/AQVN domain has a greater chance to inhibit M2 function than compounds with any other EIIP/AQVN values. The proposed AQVN/EIIP interval for the selection of dual M2 inhibitor candidates encompasses only 3% of all chemical molecules. It is therefore not surprising that the previous results from the high-throughput screening had a 10- to 100-fold lower hit rate compared to screens for other targets (Balgi et al., 2013). These results confirm that the M2 is a challenging target for selective inhibition and drug development. In the course of further analysis in our study, the next following two filters were applied in selection of candidates with dual inhibition against M2 WT and M2 S31N mutant protein. First, by applying ligand based virtual screening, the candidates were selected using lowest distance from centroid in the PCA model. This model was based on variables constructed from MIF descriptors of compounds from the learning set. Therefore, their pharmacophore similarity was criteria for the selection. Structure-based approach, as the next step, allows the docking of selected compounds from the first step into both crystal structures of M2 WT and S31N mutant proteins. In this step, the output docking energies (binding free energies) were used as criteria to rank the candidates. In order to address candidate compounds in more detail, the affinity ratio was also calculated. Two of the best candidates are presented in Figures 2, 3, and in Tables 2, 3. The biological significance of docking energy is to select the best candidate that targets both the WT and S31N mutant form of M2. i.e., a dual target candidate. We carried out docking energy comparisons for all candidates, and calculated their ratio with prior conversion to Ki values (docking energy has logarithmic dependency of Ki). Ratio of equilibrium constants between two equilibrium systems (in our case, WT and S31N mutant M2 receptors) that share same ligand in the same environment gives information about that ligand's preference to a certain receptor type. The closer the value of Ki ratio is to 1, the more the ligand is prone to target both receptor types equally and therefore, the ligand is a better dual target candidate. It should also be emphasized that it was assumed that the selected dual inhibitors targeting both M2 proteins might have advantages over mono inhibitors. This is reflected in a higher genetic barrier that enables dual inhibitors to preserve activity if the mutant reverts back to WT sequence (Ma et al., 2016; Wang, 2016).

**Figure 4:**
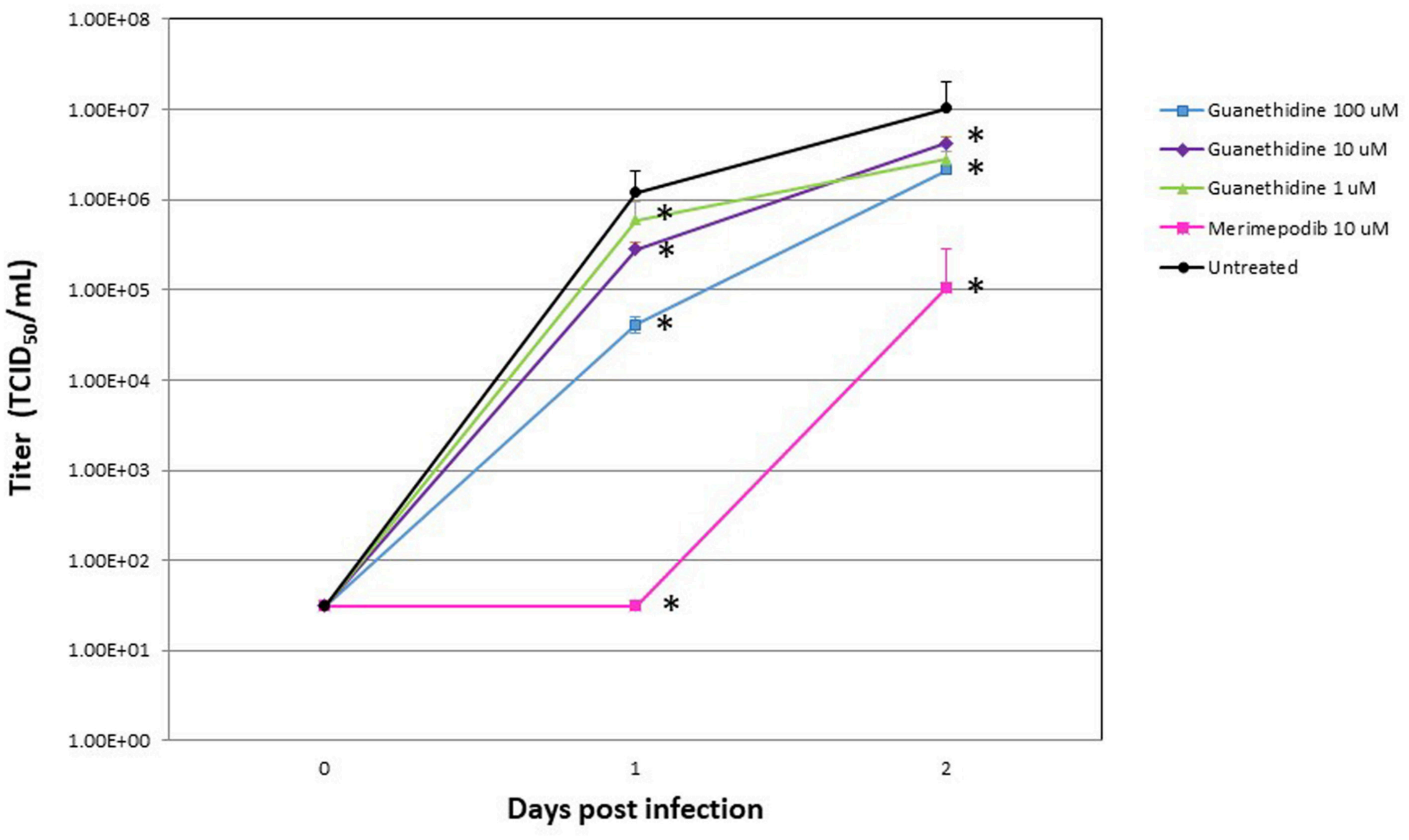
Influenza A/CA/07/2009 (H1N1) viral titers at 0, 1, and 2 days post-infection (dpi) after treatment with the indicated drug concentrations. Ten micromolar (10 μM) merimepodib was used as a positive control. Results are plotted as the means of triplicate observations, with standard deviations shown. Significant decrease in viral load is marked by asterisk.

We selected guanethidine as the best ranked compound from ligand-based virtual screening for further experimental validation. In the experiments with influenza A/CA/07/2009 (H1N1) it was shown that guanethidine inhibits influenza virus production (Figure 4) in a dose-dependent manner.

Another of the best ranked inhibitors from our computational study, methamphetamine, has actually documented good inhibitory activity against influenza A (Chen et al., 2012). It was previously demonstrated that methamphetamine inhibits influenza A virus replication *in vitro* primarily *via* acting at the viral replication stage in which M2 plays a major role. Another drug among the best ranked candidates for repurposing against M2 is cycrimine, a drug used to reduce levels of acetylcholine to balance levels of dopamine in the treatment of Parkinson's disease (Kafer and Poch, 1957; Fahn, 2015). Interestingly, the anti-influenza drug amantadine, previously repurposed for treatment of Parkinson's disease, also causes anticholinergic-like side effects (Horstink et al., 2006). In addition, as amantadine and cycrimine are in the same EIIP/AQVN domain, it can be expected from previous studies that they share same therapeutic targets. Other drugs selected as potential M2 inhibitors are ergocalciferol, calcidiol, alfacalcidol. This result is very interesting as vitamin D metabolites were previously connected to potential anti-influenza activity (Gruber-Bzura, 2018). Another FDA approved drug with documented anti-influenza properties, Alpha-Linolenic Acid, was also selected as a potential M2 inhibitor in our study, indicating the usefulness of the proposed screen (Bai et al., 2012).

In conclusion, the results presented here suggest that guanethidine represents a promising molecular template for further development of drugs against influenza virus. Other selected drugs from our computational study present valuable starting points for further experimental investigations in a quest for safe, new treatments for human and animal influenza infections.

## Data Availability

All datasets generated for this study are included in the manuscript and/or the Supplementary Files.

## Author Contributions

SP, SG, DR, and VV conceived and designed the study. VP developed the analysis tools. DR, SG, MS, VV, NV, JP, EM, and NB analyzed the data. EM and NB performed the experiments. DR, MS, VP, NV, JP, VV, EM, NB, SP, and SG drafted the work. SG, DR, VV, and SP wrote the paper. DR, MS, VP, NV, JP, VV, EM, NB, SP, and SG agreed on the final approval of the manuscript to be published and agreed to be accountable for all aspects of the work.

### Conflict of Interest Statement

The authors declare that the research was conducted in the absence of any commercial or financial relationships that could be construed as a potential conflict of interest.

## Supplementary Material

Supplementary Table 1 The learning set (M2 WT).

Supplementary Table 2 The learning set (M2 S31N).
